# Neuromelanin granules of the *substantia nigra*: proteomic profile provides links to tyrosine hydroxylase, stress granules and lysosomes

**DOI:** 10.1007/s00702-022-02530-4

**Published:** 2022-07-19

**Authors:** Maximilian Wulf, Katalin Barkovits, Karin Schork, Martin Eisenacher, Peter Riederer, Manfred Gerlach, Britta Eggers, Katrin Marcus

**Affiliations:** 1 Medizinisches Proteom-Center, Medical Faculty, Ruhr-University Bochum, Bochum, Germany; 2 Medical Proteome Analysis, Center for Proteindiagnostics (PRODI), Ruhr-University Bochum, Bochum, Germany; 3 Center of Mental Health, Clinic and Policlinic for Psychiatry, Psychosomatics and Psychotherapy, University Hospital Wuerzburg, Margarete-Hoeppel-Platz 1, 97080 Wuerzburg, Germany; 4 Psychiatry Department of Clinical Research, University of Southern Denmark Odense University Hospital, Odense C, Denmark; 5 Center of Mental Health, Department of Child and Adolescent Psychiatry, Psychosomatics and Psychotherapy, University Hospital of Wuerzburg, University of Wuerzburg, Wuerzburg, Germany

**Keywords:** Neuromelanin granules, *Substantia nigra pars compacta*, Laser microdissection, Mass spectrometry, Stress granules, Neuromelanin synthesis

## Abstract

Neuromelanin is a black-brownish pigment, present in so-called neuromelanin granules (NMGs) in the cell bodies of dopaminergic neurons in the *substantia nigra* (SN) *pars compacta*. These neurons are lost in neurodegenerative diseases, such as Parkinson’s disease and dementia with Lewy bodies. Although it is known that lipids, proteins, and environmental toxins accumulate in NMGs, the function of NMGs has not yet been finally clarified as well as their origin and the synthesis of neuromelanin. We, therefore, isolated NMGs and surrounding SN tissue from control patients by laser microdissection and analyzed the proteomic profile by tandem mass spectrometry. With our improved workflow, we were able to (1) strengthen the regularly reported link between NMGs and lysosomes, (2) detect tyrosine hydroxylase to be highly abundant in NMGs, which may be related to neuromelanin synthesis and (3) indicate a yet undescribed link between stress granules (SGs) and NMGs. Based on our findings, we cautiously hypothesize, that SGs may be the origin of NMGs or form in close proximity to them, potentially due to the oxidative stress caused by neuromelanin-bound metals.

**Supplementary Information:**

The online version contains supplementary material available at 10.1007/s00702-022-02530-4.

## Introduction

Neuromelanin (NM) is a black-brownish pigment, mainly observable in the cell bodies of dopaminergic neurons in the *substantia nigra* (SN) *pars compacta*. In the human brain, NM is stored in membrane-surrounded neuromelanin granules (NMGs) in association with proteins and lipids. These NMGs are first observable in infants while increasing in number and density of pigmentation during aging (Halliday et al. 2006). Investigating the molecular composition of NMGs is of high relevance, since the selective loss of NMG-containing dopaminergic neurons in the SN leads to cardinal symptoms in neurodegenerative diseases, such as Parkinson’s disease (PD) and dementia with Lewy bodies (DLB). Therefore, robust and reproducible OMICs techniques are of immense value, enabling the study of genes, transcripts, proteins and lipids. Nevertheless, as NMGs are absent in common laboratory animals (Marsden 1961), researchers rely on human *post mortem* brain tissue for their studies, which are limited as only a static view of NMG composition can be observed. Thus, the mechanism of NM synthesis as well as the origin of NMGs have not been finally clarified (Zecca et al. 2001; Vila 2019)**.** Concerning the NM synthesis, several hypotheses have been raised:

1. Due to similarities between NM and melanin present in human skin, one hypothesis believes the enzyme tyrosinase, responsible for melanin synthesis (Raper 1928; Mason 1965; Sánchez-Ferrer et al. 1995), to play an essential role in NM synthesis (Carballo-Carbajal et al. 2019). However, so far, only tyrosinase–mRNA could be detected and no evidence was found for the presence of tyrosinase protein in human SN tissue (Miranda et al. 1984; Xu et al. 1997; Ikemoto et al. 1998; Tief et al. 1998; Tribl et al. 2007).
2. A second enzyme believed to potentially play a role in NM synthesis is tyrosine hydroxylase (TH), the rate-limiting enzyme in the catecholamine–synthesis pathway, as NM is mainly restricted to brain regions containing catecholaminergic neurons (Saper and Petito 1982; Gaspar et al. 1983; Halliday et al. 1988; Hirsch et al. 1988). Indeed, the presence of TH protein in human brain tissue was reported in several publications (Mogi et al. 1988; Nagatsu et al. 2019), as TH is commonly used as a marker for dopaminergic neurons in immunological stainings (Robert and Meghan 2012). Nevertheless, TH is also present in the SN of rodents, such as mice and rats, in which no production of NM can be observed (Marsden 1961; Aumann et al. 2011). Even TH-overexpression did not lead to the induction of NM synthesis in mice (Mor et al. 2017).
3. A third hypothesis assumes an enzyme-free mechanism in which autooxidation of dopamine is expected to be the main process in NM synthesis (Fornstedt et al. 1986; Sulzer and Zecca 2000; Zucca et al. 2017). However, based on this assumption, a formation of NM should be observable in the SN of rats and mice as well.

Furthermore, first studies elucidating the molecular composition of NMGs on the level of proteins resulted in hypotheses on their potential origin. In-depth proteomic characterization, utilizing either density gradient purification via ultracentrifugation (Tribl et al. 2006; Zucca et al. 2018) or selective enrichment via laser microdissection (Plum et al. 2016), revealed an increased abundance of lysosomal proteins. These findings led to the assumption that NMGs may play a protective role in the human brain shielding toxic compounds, such as metals and environmental toxins from the cytoplasm (D'Amato et al. 1986; Lindquist et al. 1988; Aime et al. 1994; Zecca et al. 1996; Bridelli et al. 1999; Double et al. 2003; Tribl et al. 2006; Bohic et al. 2008; Biesemeier et al. 2016; Plum et al. 2016; Zucca et al. 2018). Other studies state that this neuroprotective role may transition ultimately resulting in neurotoxicity, as the NMG composition of highly toxic products can lead to neuroinflammation when released (Oberländer et al. 2011; Zhang et al. 2011; Cebrián et al. 2014). Therefore, the origin and role of NMGs are still widely discussed in the scientific community and need further clarification, as well as the synthesis of NM.

To gain insights into these important aspects of NMG biology, an in-depth characterization of NMGs and SN tissue surrounding NMGs (hereafter called SN_Surr._) is indispensable. Therefore, we established a revised and optimized version (Wulf et al. 2021) of our previously published workflow (Plum et al. 2016), optimizing NMG collection and in-depth proteomic characterization, leading to the identification of 170% more proteins and an improved quantification of protein abundances. In total, we were able to identify 2693 proteins in all of our measured samples of SN_Surr._ tissue and NMGs.

Thus, the present study provides the largest proteomic data set focusing on NMG biology. Concerning NM synthesis, we provide evidence for TH to be of high abundance in NMGs, while we were not able to detect tyrosinase in any of our analyzed samples. In addition, we strengthen the assumed link between NMGs and lysosomes based on their proteomic profile. For the first time, we revealed an enrichment of ribosomal and translation-associated proteins in NMGs, pointing towards a potential new aspect of their biology.

With our results, we contribute to the knowledge of NMG origin and function, opening the possibility to reconsider and extend current hypotheses.

## Materials and methods

### Ethical statement

Flash frozen, human *post mortem* SN tissue slices of 5 µm thickness were provided on 1.0 PEN-membrane glass slides (Carl Zeiss Microscopy GmbH, Göttingen, Germany) by the Navarrabiomed Biobank (Pamplona, Spain). The use of human brain tissue was approved by the ethics committee of the Ruhr-University Bochum, Germany (file number 4760-13), according to German regulations and guidelines.

### Subjects

SN tissue slices of five control (CTRL) patients, which are identified as controls based on a combination of clinical and pathological measures by the Navarrabiomed Brain Bank, were used. Additional information can be found in Table 1. SN tissue of two patients of the study cohort as well as SN tissue of two additional independent CTRL patients was used for verification experiments (Table 1, highlighted in italic).

**Table 1:** Composition of study group

Group	Sex	Age (in years)	Ø Age ± SD	PMI (h)	Ø PMI (h) ± SD
Control (*n* = 5)	Male	91	78.8 ± 12.2	4:00	4:23 ± 2:01
	Male	66		8:00	
	Female	66		3:20	
	*Female*	*82*		*3:20*	
	*Female*	*89*		*3:20*	
Control Verification (*n* = 2)	*Male*	*79*	75.5 ± 4.9	*11:00*	10:00 ± 1:25
	*Male*	*72*		*9:00*	

*Ø Age ± SD* average age and standard deviation; *PMI* *post mortem* interval, *Ø PMI (h) ± SD* average *post mortem* interval and standard deviation. Samples from patients highlighted in italic were used for verification experiments

### Laser microdissection and sample preparation

The SN tissue slices were stored at − 80 °C until further use. The isolation of NMGs and SN_Surr._ tissue was performed as previously reported (Wulf et al. 2021). In brief, NMGs covering a tissue area of 500,000 µm^2^ were isolated at 400 x-magnification and collected in water-filled caps of non-adhesive microtubes (MicroTube 500, Carl Zeiss Microscopy GmbH) using a PALM Micro Beam (P.A.L.M.-System, Carl Zeiss Microscopy GmbH). Subsequently, SN_Surr._ tissue was selected at 50 x-magnification and an area of 1,000,000 µm^2^ was collected in a fresh tube. All samples were stored at -80 °C until further use.

### Sample preparation

The sample preparation was performed as previously reported (Wulf et al. 2021). Briefly, samples were treated with formic acid (FA) and sonicated. After a drying step in a vacuum-centrifuge (Concentrator plus, Eppendorf AG, Hamburg, Germany), samples were refilled with ammonium bicarbonate, reduced with dithiothreitol and acetylated with iodoacetamide. Trypsin was added to the samples according to the collected tissue area and digestion was performed at 37 °C overnight. The digestion was stopped by adding trifluoroacetic acid (TFA). At last, samples were vacuum-dried and peptides were stored in 20 µl of 0.1% TFA at -80 °C.

### Mass spectrometric analysis

For liquid-chromatography tandem mass spectrometry (LC–MS/MS) analysis, peptide samples were diluted in 0.1% TFA. To ensure an identical peptide load, the volume of peptide solution was adapted according to the amount of collected tissue. As for each NMG sample 500,000 µm^2^, and for each SN_Surr._ sample 1,000,000 µm^2^ of tissue were collected, 5 µl of peptide solution were used for mass spectrometric measurements of NMG samples, while 2.5 µl were used for the measurements of the SN_Surr._ samples. Mixtures of all samples were measured over the course of all LC–MS/MS measurements to check for technical variances and total ion chromatogram as well as basepeak intensities were checked to ensure identical sample load. The LC–MS/MS measurements were performed on an Ultimate 3000 RSLC nano LC system (Dionex, Idstein, Germany) coupled to an Orbitrap Fusion Lumos Tribrid mass spectrometer (Thermo Fisher Scientific). At first, peptides were loaded on a pre-column (Acclaim PepMap nanoViper, Thermo Fisher Scientific; 100 µm × 2 cm, 5 µm particle size) and washed for 7 min with 0.1% TFA at a flow rate of 30 µl/min. Subsequently, the pre-column was connected to an analytical C18 column (Acclaim PepMap nanoViper, Thermo Fisher Scientific; 75 µm × 50 cm, 2 µm particle size). Separation of peptides was performed at a flow rate of 400 nL/min with a gradient starting with 95% solution A (0.1% FA) and 5% solution B (84% acetonitrile, 0.1% FA). The concentration of solution B was increased up to 30% after 105 min, then within 2 min to 95% and maintained at that level for 3 further minutes. Subsequently, the column was adjusted back to 5% solution B. These gradient settings were used for data dependent acquisition (DDA)-experiments, as well as for parallel reaction monitoring (PRM)-experiments. The nano LC-system was directly coupled to the electrospray ionization source (Thermo Fisher Scientific) of the Orbitrap Fusion Lumos Tribrid mass spectrometer.

#### Global mass spectrometry

For global DDA-experiments, the system operated within a scan range from 350 to 1400 m/z with a resolution of 120,000 and a maximum injection time of 80 ms. In a fixed cycle time of 2 s, all precursor ions with an intensity above 1 × 10^4^ were selected for fragmentation at a fixed collision energy of 28% by higher energy collisional dissociation (HCD). Precursor ions selected for fragmentation were maintained on a dynamic exclusion list for 30 s. Fragment ion scans were performed at a resolution of 30,000 with a maximum injection time of 80 ms.

#### Targeted mass spectrometry

For PRM-experiments, the system operated within a scan range of 350 to 1400 m/z with a resolution of 120,000 and a maximum injection time of 50 ms. An inclusion list containing 7 precursor ions, representing unique peptides of tyrosine hydroxylase, glycerol-3-phosphate dehydrogenase and cytoplasmic dynein 1 heavy chain 1 was applied (Table 2). For each peptide precursor, a retention time (RT) window of 3 min was defined in the inclusion list. During the RT window, the peptide precursor was selected by the Quadrupole for fragmentation at a collision energy of 28% by HCD. The fragment ion scan was carried out in the Orbitrap at a resolution of 60,000 with a maximum injection time of 200 ms.

**Table 2:** Peptides selected for parallel reaction monitoring-experiments

Protein	Peptide	m/z	Charge state	RT-window mean [± 1.5 min]
Tyrosine hydroxylase	AGGPHLEYFVR	415.884	3 +	43.4
	FDPDLDLDHPGFSDQVYR	712.6605	3 +	75.6
	TGFQLRPVAGLLSAR	529.3106	3 +	69.8
Glycerol-3-phosphate-dehydrogenase	NHVVDISESGLITIAGGK	603.995	3 +	67.3
	LAFLNVQAAEEALPR	821.4516	2 +	89.5
Cytoplasmic dynein 1 heavy chain 1	ESPEVLLTLDILK	735.4267	2 +	105.6
	EFGPVVIDYGK	612.319	2 +	66.1

*m/z* mass-to-charge ratio, *RT* retention time

The mass spectrometry proteomics data have been deposited to the ProteomeXchange Consortium via the PRIDE (Perez-Riverol et al. 2022) partner repository with the data set identifier PXD033533.

### Data analysis and statistics

#### Global mass spectrometry

The MaxQuant software (version 1.6.17.0) was used for the analysis of raw files. The resulting peak lists were searched against the human UniProt FASTA reference proteome (version 20th January 2021; 75,796 entries) and a common contaminants database provided in the Andromeda search engine (Cox and Mann 2008; Cox et al. 2011; Tyanova et al. 2016a). Due to sample preparation, carbamidomethlyation of methionine was set as fixed modification and carbamidomethylation of any N-terminus, oxidation of methionine as well as deamidation of asparagine and glutamine were set as variable modifications. Trypsin was set as digestion enzyme and a maximum number of 2 missed cleavages were tolerated in the search. The false discovery rate (FDR) was set to 1% for peptides (minimum length of seven amino acids) and proteins and was determined by searching against a reverse decoy database. Peptide identification was carried out with an initial allowed precursor mass deviation up to 5 parts per million (ppm) and an allowed fragment mass deviation of 20 ppm. Match between runs was enabled to enhance and optimize peptide identification. Label free quantification (LFQ) was performed using the MaxQuant LFQ normalization, whereby the Top10 unique and razor peptides were assessed for protein quantification (Cox et al. 2014). In addition, intensity based absolute quantification (iBAQ) value calculation was enabled. IBAQ values were normalized for each sample and multiplied by 1000 to obtain per mille values. Of these normalized iBAQ values, mean values were created for each sample group (NMG, SN_Surr._). Protein groups (hereafter referred to as proteins) with normalized mean iBAQ values > 0 were considered as identified.

The resulting protein group output was analyzed using the Perseus software (version 1.6.15.0), as part of the MaxQuant environment (Tyanova et al. 2016b), whereby master mixes were excluded for the subsequent analysis. Contaminants and decoys were filtered out and LFQ values were subsequently log2 transformed for further statistical analysis. A minimum of 70% valid values per group was set as an additional filtering criterion prior to relative quantification of proteins. Prior to statistical analysis, the remaining missing values were imputed from a normal distribution using a width of 0.3 and a downshift of 1.8. Paired Student’s *t* test as well as *p* value correction by Benjamini–Hochberg were applied to the data set (leading to *q* values) to determine proteins with significantly differential abundances. Proteins with a *p* value < 0.05 were defined as being significantly differential, while *q* values were applied as more stringent statistical criteria. Fold changes were determined by calculating the ratio of means between the two assessed groups (NMG, SN_Surr._).

The results of this analysis are presented as a Volcano plot, created in R version 4.0.3 (R Foundation for Statistical Computing 2020) using the packages ggplot2 3.3.3 and ggrepel 0.9.1. Negative log10-transformed *p* values (uncorrected) are plotted against the log2-transformed fold changes. Proteins that reach a corrected *p* value < 0.05 are marked in red. On each side, the ten proteins with the largest Euclidean distance from the origin in the Volcano plot are labeled with the corresponding gene name.

For principal component analysis (PCA), analysis was conducted and graphics were created in R using the same software versions as for the Volcano plot. LFQ values from MaxQuant were taken and log2-transformed, zero values were treated as missing values. Proteins with more than 50% missing values were excluded and the remaining missing values were imputed by the mean of the corresponding protein intensities. A PCA was calculated using the setting that scales the data beforehand. The first two principal components (PC) were plotted.

Differentially expressed proteins as well as identified proteins in SN_Surr._ and NMGs based on iBAQ were further analyzed by GO (gene ontology)-term and pathway enrichment analysis. In brief, proteins were used for annotation enrichment using the web version of DAVID Bioinformatics Resources 6.8 (Da Huang et al. 2009a, 2009b) to gain a first overview of differences in the proteomic profile between NMGs and SN_Surr._ tissue hinting towards characteristic functions. The *homo sapiens* proteome served as a reference and GO-terms from the categories Biological Process (BP), Cellular Compartment (CC), Molecular Function (MF) and KEGG Pathways were taken for in-depth analysis. A *p* value < 0.05 was used as a significance threshold and fold enrichment scores were taken to assess the enrichment of resulting GO-terms and pathways. To search for information on specific proteins, the UniProt database was utilized (The UniProt Consortium 2021).

#### Targeted mass spectrometry

Verification of differentially expressed proteins with significant *p* values was carried out by PRM experiments for tyrosine hydroxylase, glycerol-3-phosphate dehydrogenase and cytoplasmic dynein 1 heavy chain 1. For that, the resulting peptide output of MaxQuant was processed in an identical manner as our protein group output and peptides assigned to TH were examined for significantly differential expression (*p* value < 0.05), fold change, retention time and charge state. As housekeeping proteins, glycerol-3-phosphate dehydrogenase and cytoplasmic dynein 1 heavy chain 1 were chosen, as they were found to be neither significant nor differential between NMGs and SN_Surr._ tissue and additionally displayed a low standard deviation on the level of intensities. Evaluation of PRM data was performed using Skyline software (MacLean et al. 2010). Group comparisons were performed on peak areas for each protein and an adjusted *p* value < 0.05, according to Benjamini–Hochberg, was used as a significance threshold on MS2-level.

## Results

### Global proteomic characterization of NMGs and surrounding tissue

**Fig. 1:**
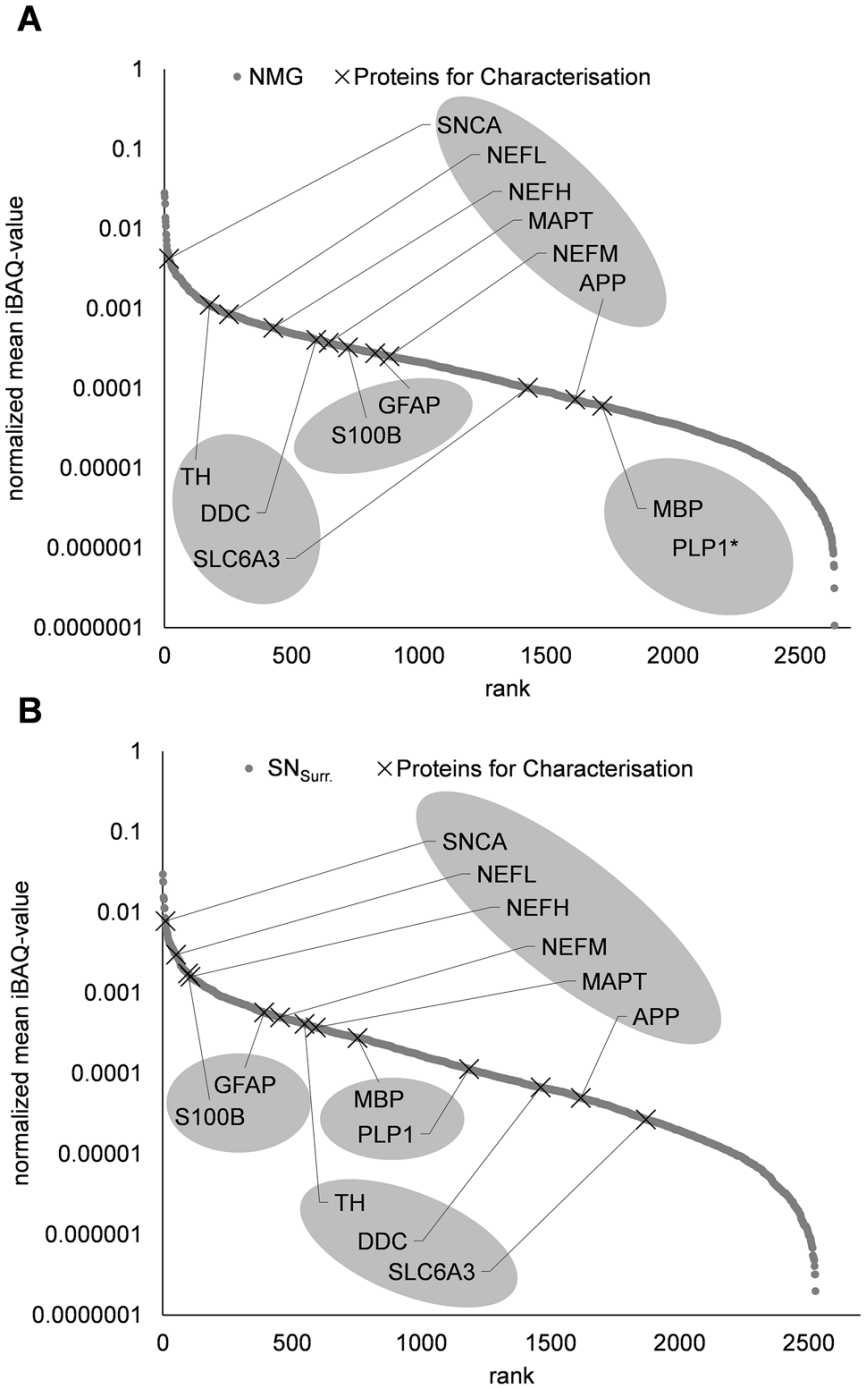
Ranking plots of protein abundances in NMGs and surrounding tissue. Plots show ranked normalized mean iBAQ-values of all identified proteins in both sample groups (NMG, SN_Surr._). **A** Neuronal and dopaminergic markers rank highest in NMGs, while markers for astrocytes and oligodendrocytes are relatively low in abundance. PLP1 was not identified in NMG samples. **B** Compared to NMGs, astrocytic markers are higher abundant in SN_Surr._ tissue, while dopaminergic markers are lower abundant. Oligodendrocyte markers are higher abundant compared to NMGs. *SNCA* alpha-synuclein, *NEFL* neurofilament light chain, *NEFM* neurofilament medium chain, NEFH: neurofilament heavy chain, APP: amyloid beta precursor protein, *MAPT* microtubule associated protein tau, *TH* tyrosine hydroxylase, *DDC* aromatic-L-amino-acid decarboxylase, *SLC6A3* solute carrier family 6 member 3, *GFAP* glial fibrillary acidic protein, *S100B* S100 calcium binding protein B, *MBP* myelin basic protein, *PLP1* proteolipid protein 1

We first set out to characterize the individual protein profiles of NMGs and SN_Surr._ tissue (Fig. 1, Supplementary Table 1). As the main components of brain tissue are neurons (in case of SN tissue dopaminergic neurons), oligodendrocytes and astrocytes, we confirmed the high abundance of selected marker proteins for each cell type in NMGs and SN_Surr._ tissue (Fig. 1, neurons: alpha-synuclein (SNCA), neurofilament light (NEFL), medium (NEFM) and heavy chain (NEFH), amyloid beta precursor protein (APP), microtubule associated protein tau (MAPT); dopaminergic neurons: tyrosine hydroxylase (TH), aromatic-L-amino-acid decarboxylase (DDC), solute carrier family 6 member 3 (SLC6A3); astrocytes: glial fibrillary acidic protein (GFAP), S100 calcium binding protein B (S100B); oligodendrocytes: myelin basic protein (MBP), proteolipid protein 1 (PLP1)).

Especially neuronal and astrocytic markers such as SNCA, NEFL, S100B, NEFH and GFAP were found to be highly abundant in SN_Surr._ tissue, indicating the presence of astrocytes and neurons in the tissue surrounding NMGs, whereas the dopaminergic marker proteins DDC and TH were of lower abundance.

DDC and TH were instead found to be of higher abundance in NMGs, while both astrocytic markers, GFAP and S100B, were found to be lower abundant in NMGs. Neuronal markers SNCA, NEHL and NEFH showed a similar abundance pattern as in SN_Surr._ tissue. Marker proteins for oligodendrocytes showed low abundance levels in both sample types, PLP1 was not even identified in NMGs. These findings support the localization of NMGs in cell bodies of dopaminergic neurons and allow the characterization of the SN_Surr._ tissue as mainly composed of neurons and astrocytes, while oligodendrocytes play only a minor role.

### NMGs and surrounding tissue can be separated based on their proteomic profile

As the majority of proteins present in the human brain are not restricted to a single cell type or cell compartment, a proteomic study based only on the identification of proteins may miss important information about NMGs. Therefore, we also included the abundances of proteins in our analysis to gain information about proteins being enriched in either NMGs or SN_Surr._.

**Fig. 2:**
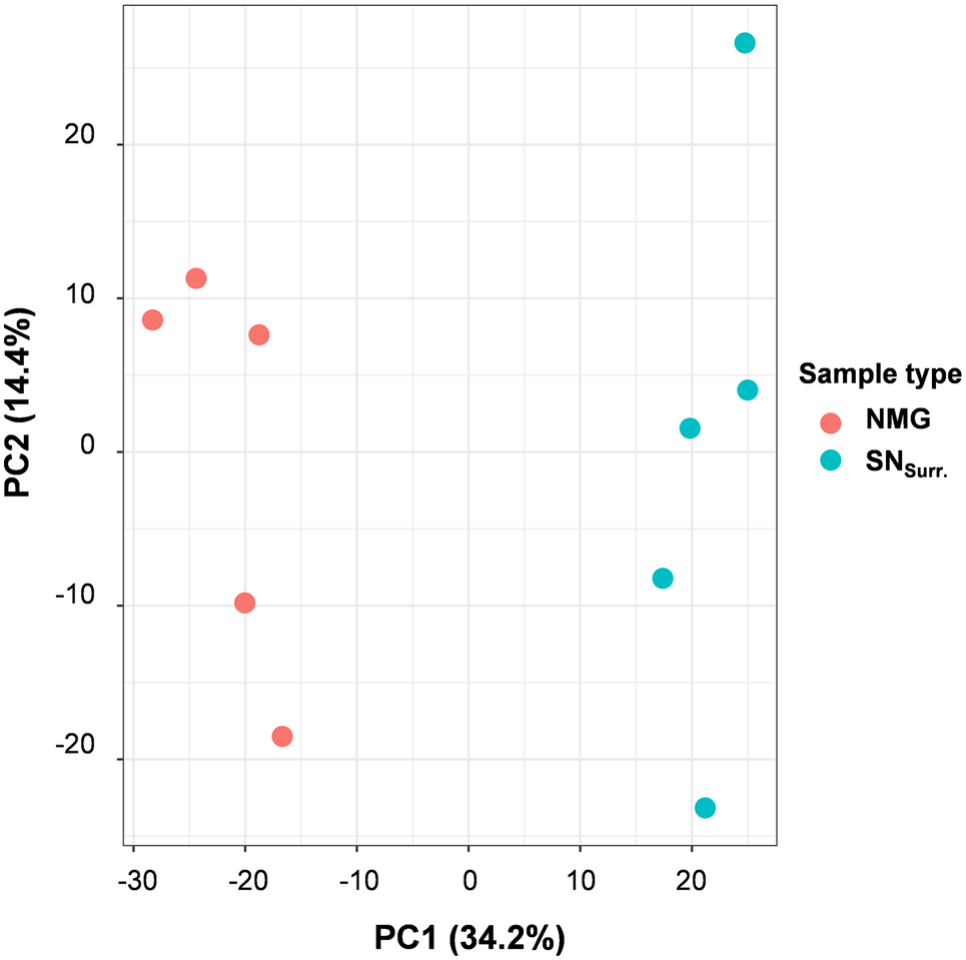
Principal component analysis (PCA). The measured samples form two clusters based on the sample groups (NMG, SN_Surr._) on Principal Component 1 (PC1)

Our data set included 1088 proteins, which suited the criteria for our quantitative comparison (Supplementary Table 2). As the principal component analysis (PCA) in Fig. 2 shows, NMGs and SN_Surr._ can be distinguished already with a high percentage on Principal Component 1, exceptionally stressing their high individuality on the proteome level.

### Differential proteins hint towards an involvement of NMGs in protein synthesis

**Fig. 3:**
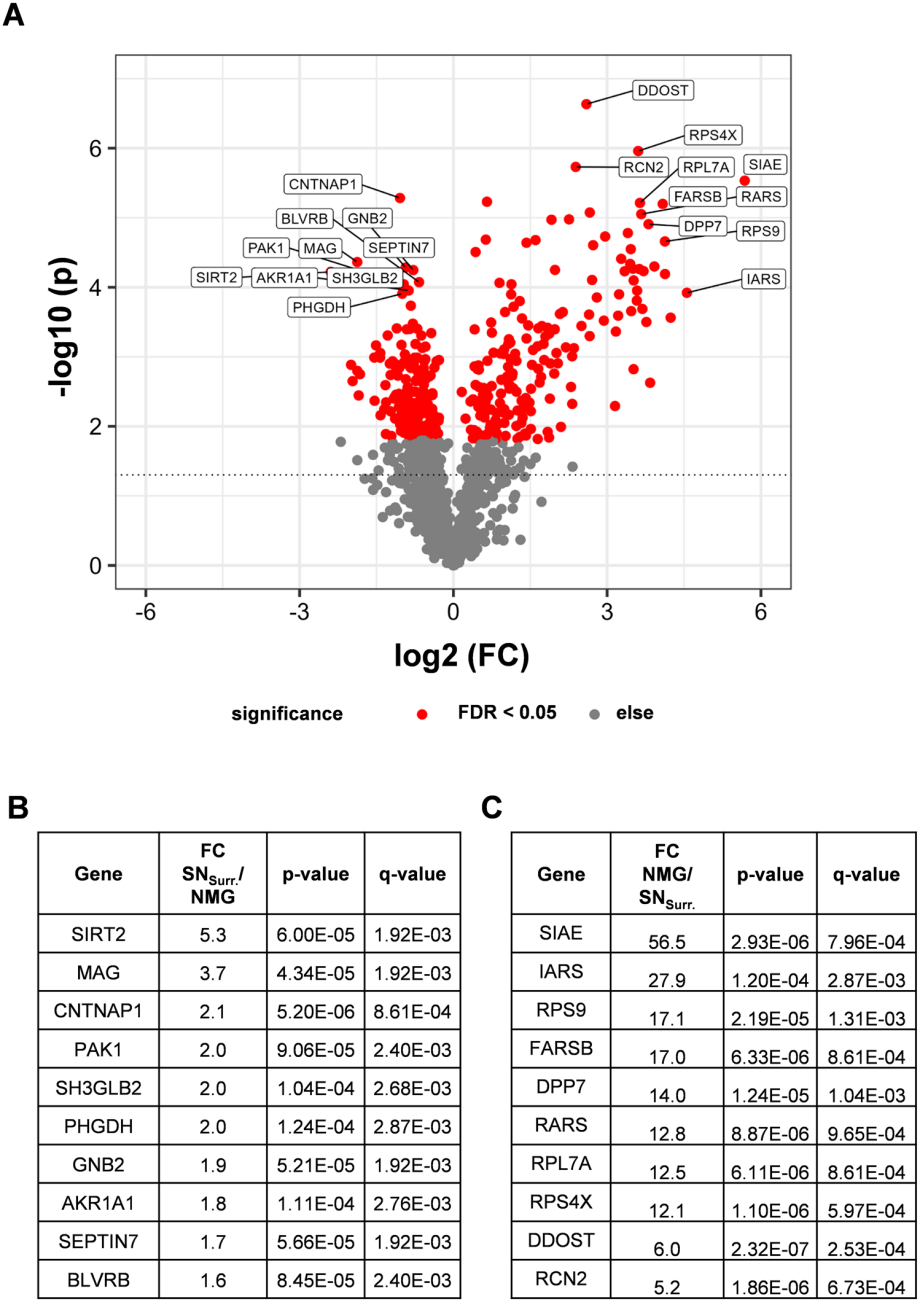
Volcano plot showing the relationship between fold change (FC) and *p* value for the comparison of protein abundance in NMGs and SN_Surr._ tissue. **A** Red dots indicate proteins with a significant *q* value (< 0.05). The 10 proteins with the highest Euclidean distances in both FC directions are annotated with their gene names and FC, *p* and *q* values are displayed in the tables shown in **B** and **C**. *SIRT2* NAD-dependent protein deacetylase sirtuin-2, *MAG* myelin-associated glycoprotein, *CNTNAP1* contactin-associated protein 1, *PAK1* serine/threonine–protein kinase PAK 1, *SH3GLB2* endophilin-B2, *PHGDH* D-3-phosphoglycerate dehydrogenase, *GNB2* guanine nucleotide-binding protein G(I)/G(S)/G(T) subunit beta-2, AKR1A1: alcohol dehydrogenase [NADP( +)], *SEPTIN7* Septin-7, *BLVRB* flavin reductase (NADPH), *SIAE* sialate O-acetylesterase, *IARS* isoleucine–tRNA ligase, *RPS9* 40S ribosomal protein S9, *FARSB* phenylalanine–tRNA ligase beta subunit, *DPP7* dipeptidyl peptidase 2, *RARS* arginine–tRNA ligase, *RPL7A* 60S ribosomal protein L7a, *RPS4X* 40S ribosomal protein S4 X isoform, *DDOST* dolichyl-diphosphooligosaccharide–protein glycosyltransferase 48 kDa subunit, *RCN2* reticulocalbin-2

Since especially proteins with high and significant fold changes may provide insights into characteristics of NMGs and SN_Surr._ tissue, we focused especially on these proteins when evaluating the results of our quantitative comparison. Indeed, 345 proteins showed a statistically significant differential abundance (adjusted *p* value < 0.05), displayed in the Volcano Plot (Fig. 3). For further in-depth characterization, we focused on the Top 10 proteins (Fig. 3B, C) regarding high and significant fold changes.

Among these proteins, lysosomal sialate O-acetylesterase (SIAE) showed the highest enrichment in NMGs (Fig. 3C), strengthening the relationship between lysosomes and NMGs. Furthermore, a link to protein synthesis was found, since three tRNA ligases (isoleucine–tRNA ligase (IARS), phenylalanine–tRNA ligase beta subunit (FARSB), arginine–tRNA ligase (RARS)) and three ribosomal proteins (40S ribosomal protein S4 X isoform (RPS4X), 60S ribosomal protein L7a (RPL7A), 40S ribosomal protein S9 (RPS9)) showed higher levels in NMGs.

In contrast, the top ten proteins enriched in SN_Surr._ tissue (Fig. 3B) were found to be characteristic for neuron-surrounding tissue, such as the myelin-associated glycoprotein (MAG) as a part of the neuronal myelin sheath. Proteins exclusively identified in SN_Surr._ tissue stressed the neuronal character of the tissue, as for example myelin proteolipid protein, dynein light chain 1 and peripherin were included in this list (Supplementary Table 1).

In summary, our quantitative comparison strengthens the previously reported relation between lysosomes/endosomes and NMGs (Plum et al. 2016; Zucca et al. 2018). Nevertheless, as proteins associated with protein synthesis were found to be significantly higher abundant in NMGs, our analysis may open a new perspective of NMG biology as, to our knowledge, enhanced abundances of ribosomal, mRNA-binding and translation-related proteins in NMGs were never reported before. This finding is supported by the results of a GO-term analysis of proteins exclusively identified in NMGs, which revealed a variety of proteins to be related to ribosomes, mRNA-binding and translation (Supplementary Table 1).

### Stress granule-related proteins are significantly higher abundant in NMGs

Especially, the high abundances of proteins of the 40S ribosomal subunit in combination with several eukaryotic translation initiation factors (eIF) and RNA-binding proteins raised the question of a potential link between NMGs and stress granules (SGs). SGs are transient structures, which form after the inhibition or interruption of translation and, therefore, contain the aforementioned protein species (Kedersha et al. 2016).

**Fig. 4:**
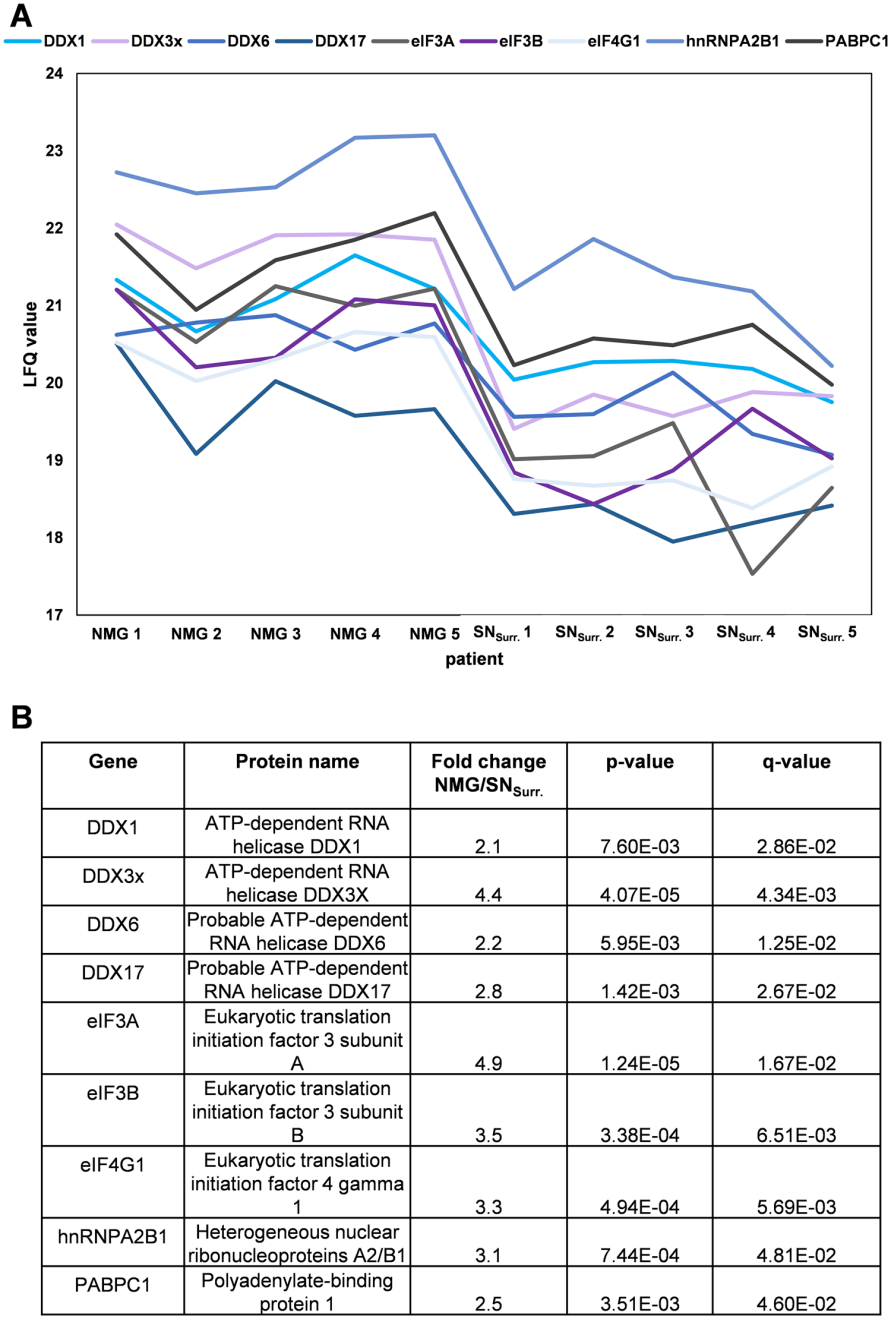
Summary of the LFQ-based quantitative comparison for nine stress granule marker proteins. **A** LFQ-values for nine stress granule marker proteins are higher in NMGs (left) than in SN_Surr._ tissue (right) for all five patients included in this study showing the higher protein abundances. **B** Fold changes between NMGs and SN_Surr._ are rather mild, but the differences are significant after Benjamini–Hochberg correction. *DDX1* ATP-dependent RNA helicase DDX1, *DDX3x* ATP-dependent RNA helicase DDX3X, *DDX6* probable ATP-dependent RNA helicase DDX6, *DDX17* probable ATP-dependent RNA helicase DDX17, *eIF3A* eukaryotic translation initiation factor 3 subunit A, *eIF3B* eukaryotic translation initiation factor 3 subunit B, *eIF4G1* eukaryotic translation initiation factor 4 gamma 1, *hnRNPA2B1* heterogeneous nuclear ribonucleoproteins A2/B1, *PABPC1* polyadenylate-binding protein 1

To further characterize this potential link, we analyzed the results of our quantitative comparison with a special focus on SG-specific proteins based on a previously published list of 37 proteins (Wolozin and Ivanov 2019). Nine well-known SG proteins, amongst them polyadenylate-binding protein 1 (PABPC1), which is frequently used in immunohistochemical stainings as a marker protein for SGs, were significantly higher abundant in NMGs with fold changes between 2.1 and 4.9-fold (Fig. 4).

Due to the strict filter criteria for our quantitative comparison, we also checked our data set for the global proteome analysis. In this, 23 additional SG-specific proteins were included, all of them showing higher abundances in NMGs compared to SN_Surr._ tissue. In addition, three SG-proteins (Caprin 1, Caprin 2 and probable ATP-dependent RNA helicase DDX5) were exclusively identified in NMGs (Supplementary Table 1).

In summary, our complete data set contains data for 32 of the previously published 37 SG-proteins, of which all are higher abundant in NMGs. Through our quantitative comparison, we provide clear evidence for elemental components of SGs (Kedersha et al. 2005) such as polyadenylate-binding protein 1 (PABPC1), subunits of eukaryotic translation initiation factor 3 (eIF3A, eIF3B) and eIF4 gamma 1 (eIF4G1) being significantly higher abundant in NMGs than in surrounding tissue.

### Tyrosine hydroxylase is significantly higher abundant in NMGs

Since the findings presented up to this point were primarily the result of a systematic comparison, we wanted to take another look at those aspects of NMG biology that have already been intensively studied in the past, namely, the involvement in the iron metabolism of the SN and the mechanism of NM synthesis.

Stressing the importance of iron metabolism in the SN, ferritin heavy chain was amongst the highest abundant proteins in both NMGs and SN_Surr._ tissue, with a significantly higher abundance in SN_Surr._ tissue (1.8-fold, Supplementary Tables 1, 2), while ferritin light chain was comparably abundant in both of our sample groups as well as serotransferrin.

Concerning NM synthesis, we focused on proteins involved in dopamine metabolism as well as tyrosinase, since it is often assumed to be involved in NM synthesis. Although a highly sensitive state-of-the-art mass spectrometer was used, tyrosinase was not detected in any of our measurements, neither in SN_Surr._ tissue, nor in NMGs. For proteins involved in dopamine metabolism, we focused on tyrosine hydroxylase (TH) as the rate limiting enzyme in catecholamine biosynthesis and, therefore, essential in the synthesis of dopamine, as well as aromatic-L-amino-acid decarboxylase (DDC), monoamine oxidase A and B (MAOA, MAOB), vesicular monoamine transporter 2 (VMAT2/SLC18A2) and dopamine transporter (DAT/SLC6A3).

**Fig. 5:**
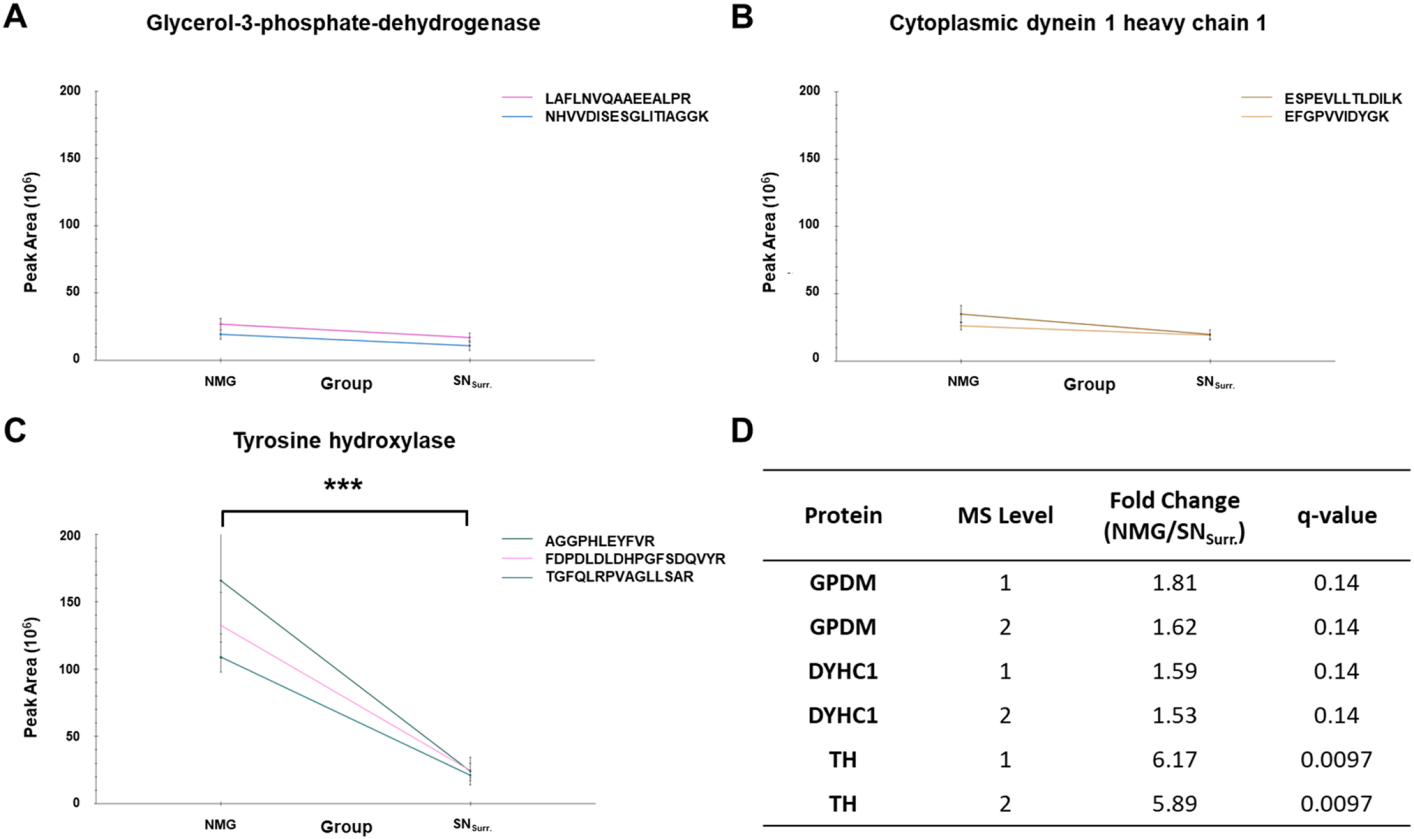
PRM results for proteins chosen for verification. **A**–**C** Graphs show intensities of selected peptides for GPDM (**A**), DYHC1 **B** and TH **C** as mean values for NMGs and SN_Surr._ tissue. Mean values for peptides of GPDM and DYHC1 are comparable, only the mean value of peptides for TH is significantly higher in NMGs compared to SN_Surr._ tissue. **D** Summary of the results of our PRM-experiments, showing an even higher fold change (~ 6) for TH than the DDA-experiments. ***: *q* value < 0.001. *GPDM* glycerol-3-phosphate dehydrogenase, mitochondrial, *DYHC1* cytoplasmic dynein 1 heavy chain 1

In our quantitative comparison, we found both proteins required for dopamine synthesis, TH (fold change: 2.5, *q* value: 0.03) and DDC (fold change: 3.3; *q* value: 0.01), to be significantly higher abundant in NMGs. We could further verify the significantly higher abundance of TH in NMGs in independent targeted MS-experiments (Fig. 5), in which only peptides unique for TH were quantified. To ensure identical sample load we used two reference proteins (Glycerol-3-phosphate dehydrogenase, mitochondrial **(**GPDM) and cytoplasmic dynein 1 heavy chain 1 (DYHC1)), thus verifiying the increased abundance of TH in NMG tissue.

Further proteins associated with dopamine metabolism did not display such a cohesive picture. Conversely, MAOA showed a comparable abundance in SN_Surr._ tissue and NMGs (fold change: 0.83; *q* value: 0.18), while MAOB was significantly higher abundant in SN_Surr._ tissue (fold change: 0.59; *q* value: 0.04).

VMAT2 was not identified at all in any of our samples. DAT instead did not match the criteria of our quantitative comparison, but protein levels showed a higher abundance in NMGs.

Summarizing our findings, we provide evidence for enzymes required for dopamine synthesis, TH and DDC, being highly abundant in NMGs.

## Discussion

Our here presented study represents the largest data set concerning the global proteome of NMGs, including 2693 identified proteins, of which 1088 were examined using a relative quantification approach allowing an in-depth characterization of protein abundances in NMGs and surrounding SN tissue for the first time. Both, identified and quantified proteins, give further insights into open questions of NMG biology concerning origin and function of NMGs as well as NM synthesis.

### Origin and function of neuromelanin granules

The origin and function of NMGs are widely discussed. With our proteomic characterization we were able to highlight several key characteristics of the proteomic compound of NMGs, potentially revealing distinct functions in the SN. Until now, the most widely accepted theory regarding NMG origin claims a close relation between NMGs and lysosomes or endosomes (Tribl et al. 2006; Plum et al. 2016; Zucca et al. 2018). This theory is based on the results of several proteomic studies and the observation that NMGs are surrounded by a double membrane, a distinct feature of organelle structures (D'Agostino and Luse 1964; Tribl et al. 2006; Plum et al. 2016; Zucca et al. 2018). In a first approach on characterizing NMGs, we (Plum et al. 2016) further hypothesized that NM may be first generated in the cytosol and then taken up by lysosomes, resulting in NMG formation.

In the present study, we can confirm the link between NMGs and lysosomes based on higher abundances of lysosomal proteins such as SIAE, Lysosome-associated membrane glycoprotein 2 (LAMP2) and several cathepsines in NMGs compared to SN_Surr._ tissue (Supplementary Tables 1, 2). In addition, the identification of typically non-neuronal proteins such as GFAP in NMGs may be another hint towards their lysosomal role. To ensure, that the presence of these proteins in our samples is not based on contaminations due to our methodological approach, we compared our data set with previously published studies focusing on the proteomic content of NMGs (Plum et al. 2016; Zucca et al. 2018). Since GFAP was identified in NMGs in both studies, although the latter one (Zucca et al. 2018) used an independent methodological approach for the purification of NMGs in density gradient purification via ultracentrifugation, this seems to be a reliable finding.

While these observations would assume that NMGs are a degradative organelle, we are the first to provide evidence for a link between SGs and NMGs, as we detected several well-known SG-associated proteins in the NMGs. From 37 SG proteins (Wolozin and Ivanov 2019), we were able to identify 32 in our data set, all of them being higher abundant in NMGs.

In general, SGs are known to form under external stress and influence mRNA function, localization as well as signaling pathways (Buchan and Parker 2009; Buchan et al. 2013; Kedersha et al. 2013; Wheeler et al. 2016). However, recent studies suggest that the frequently observed aggregates in various neurodegenerative disorders may originate from SGs, which develop into persistent protein aggregates under constant cell stress (Wolozin 2012; Dobra et al. 2018). Indeed, we could find TAR DNA-binding protein 43 (TDP43), which is the main component of protein aggregates in amyotrophic lateral sclerosis (Neumann et al. 2009), to be higher abundant in NMGs than in SN_Surr._ tissue.

Our data thus indicate that NMGs, similar to protein aggregates in neurodegenerative diseases, contain several SG marker proteins. We, therefore, cautiously raise two possible hypotheses, potentially explaining the relation between SGs and NMGs:

- 1. SGs may be the origin of NMGs as a result of chronic cell stress.
- 2. SGs form in close proximity to NMGs.

A major stress factor contributing to transient SG formation and potentially their transformation into persistent NMG structures could be oxidative stress caused by NM-bound metals (Sian-Hülsmann et al. 2011; Riederer et al. 2021). It was reported that metal concentrations in SN are high and that metal ions are associated with neuromelanin within NMGs (Zecca and Swartz 1993). In addition, we were able to show that iron-binding proteins such as ferritin light and heavy chain were highly abundant in NMGs, but also in the surrounding tissue, assuming that they are not NMG specific (Supplementary Table 1).

Still, we have to point out that NMGs, unlike SGs, are membrane-surrounded and thus may be closer related to lysosomes, since both inherit misfolded protein species and lyososomal/endosomal proteins (Tribl et al. 2005, 2006; Plum et al. 2016; Zucca et al. 2018). Unfortunately, we are not able to verify our hypotheses with the methods applied in this manuscript. To study the potential link between SGs and NMGs and to commit to one of our hypotheses, functional studies in suitable neuromelanin-like pigments producing models (Hasegawa et al. 2003; Jo et al. 2016; Carballo-Carbajal et al. 2019) will be of immense importance, for example based on immunological stainings of SG marker proteins.

### Neuromelanin synthesis

Besides origin and function of NMGs, the synthesis of NM is highly debated as well. The three most common hypotheses focus either on TH or on tyrosinase to be involved in an enzymatic synthesis or assume an autooxidative generation with dopamine being a potential precursor for NM. With our proteomics approach, we are able to provide evidence for TH to be of high abundance in NMGs, while not a single peptide belonging to tyrosinase was detected in any of our samples.

In catecholaminergic neurons, it is widely accepted that TH is localized in the neuronal cell body in the *substantia nigra*, as well as in the presynaptic axon terminals in the *striatum* (Muñoz et al. 2012). As we used *substantia nigra pars compacta* tissue for our analysis, cell bodies of dopaminergic neurons should be present, while presynaptic terminals of dopaminergic neurons should be absent. TH is essential for the production of dopamine, further such as DDC and vesicular VMAT2 are also involved in dopamine synthesis and packaging. DDC catalyzes the conversion of L-dihydroxyphenylalanine (L-DOPA) into dopamine, while the packaging into synaptic vesicles is achieved by the activity of VMAT2 (Cartier et al. 2010). VMAT2 was not identified in any of our MS-measurements, but for DDC, a significantly higher abundance in NMGs compared to SN_Surr._ tissue was revealed in our quantitative comparison. MAOA and MAOB, proteins involved in dopamine degradation (Finberg 2019), were found to be comparably abundant in SN_Surr._ tissue and NMGs instead (MAOA) or significantly higher abundant in SN_Surr._ tissue (MAOB), while immunohistochemical stainings only revealed the presence of MAOA and MAOB in glial cells surrounding NMG-containing neurons. (Konradi et al. 1988).

These findings suggest that production of dopamine may take place in NMGs of the SN, although our data cannot prove the production of dopamine by functional TH and DDC. Thus, this hypothesis needs to be further investigated in the future. Nevertheless, it is known that dopamine not imported into vesicles by VMAT2 either oxidizes into aminochrome, a precursor of neuromelanin, or can be degraded by MAOA (Smythies 1996; Muñoz et al. 2012; Goldstein 2021).

Another interesting finding in this context is the higher abundance of DAT/SLC6A3 in NMGs compared to SN_Surr._ tissue. Although the mediation of the reuptake of dopamine from the synaptic cleft is the main task of DAT, it is also reported that DAT is able to release dopamine in the extracellular space (Khoshbouei et al. 2003). In addition to its localization in the plasma membrane, DAT was observed to be localized in the soma of dopaminergic neurons at the endoplasmic reticulum, the Golgi apparatus and in “multivesicular body vesicles” (Hersch et al. 1997). Therefore, the localization of DAT in NMGs could indicate a functional role, although the direction of dopamine transport mediated by DAT in NMGs needs further examination.

However, it is important to mention that not all TH-positive neurons in the human SN contain NMGs (Hirsch et al. 1989; Saper and Petito 1982) and that no NM synthesis can be observed in mice or rats, although dopamine is synthesized in their brains.

## Conclusions

Concluding, our data provides us with further evidence on NM synthesis and NMG origin. Based on our analysis of the proteomic profile of NMGs and surrounding SN tissue, we hypothesize that SGs resulting from cellular stress may be the origin of NMGs or that they form in close proximity to NMGs. In addition, we provide further evidence for the link of NMGs and lysosomes. Concerning NM synthesis, we hypothesize that NM may be synthesized in a pathway involving TH and autooxidative processes. Still, to verify our findings, functional analyses in appropriate cell culture or animal models are inevitable to further elucidate NMG genesis and function in disease progression.

## Supplementary Information

Below is the link to the electronic supplementary material.

Supplementary file1 (XLSX 2791 KB)

Supplementary file2 (XLSX 1200 KB)
